# Ca^2+^/calmodulin kinase II–dependent regulation of β_IV_-spectrin modulates cardiac fibroblast gene expression, proliferation, and contractility

**DOI:** 10.1016/j.jbc.2021.100893

**Published:** 2021-06-18

**Authors:** Drew M. Nassal, Nehal J. Patel, Sathya D. Unudurthi, Rebecca Shaheen, Jane Yu, Peter J. Mohler, Thomas J. Hund

**Affiliations:** 1 The Frick Center for Heart Failure and Arrhythmia, The Dorothy M. Davis Heart and Lung Research Institute, The Ohio State University Wexner Medical Center, Columbus, Ohio, USA; 2 Department of Biomedical Engineering, College of Engineering, The Ohio State University, Columbus, Ohio, USA; 3 Department of Physiology & Cell Biology, College of Medicine, The Ohio State University Wexner Medical Center, Columbus, Ohio, USA; 4 Department of Internal Medicine, College of Medicine, The Ohio State University Wexner Medical Center, Columbus, Ohio, USA

**Keywords:** β_IV_-spectrin, calmodulin kinase II, cardiac fibroblast, fibrosis, STAT3, β_IV_ifKO, inducible fibroblast-specific β_IV_-spectrin knockout mouse, β_IV_-S2254E, β_IV_-spectrin with glutamic acid substitution at serine 2254 (phosphomimetic), AngII, angiotensin II, CaMKII, Ca^2+^/calmodulin-dependent kinase II, cDNA, complementary DNA, CF, cardiac fibroblast, CHX, cycloheximide, DAPI, 4′,6-diamidino-2-phenylindole, DMEM, Dulbecco's modified Eagle's medium, ECM, extracellular matrix, FBS, fetal bovine serum, FDR, false discovery rate, HA, hemagglutinin, MS/MS, tandem MS/MS, NIH, National Institutes of Health, qPCR, quantitative real-time PCR, *qv*^*3J*^, mutant β_IV_-spectrin allele C-terminal region containing CaMKII interaction motif, *qv*^*4J*^, mutant β^IV^-spectrin allele lacking repeats 10 through C terminus, STAT3, signal transducer and activation of transcription 3

## Abstract

Fibrosis is a pronounced feature of heart disease and the result of dysregulated activation of resident cardiac fibroblasts (CFs). Recent work identified stress-induced degradation of the cytoskeletal protein β_IV_-spectrin as an important step in CF activation and cardiac fibrosis. Furthermore, loss of β_IV_-spectrin was found to depend on Ca^2+^/calmodulin-dependent kinase II (CaMKII). Therefore, we sought to determine the mechanism for CaMKII-dependent regulation of β_IV_-spectrin and CF activity. Computational screening and MS revealed a critical serine residue (S2250 in mouse and S2254 in human) in β_IV_-spectrin phosphorylated by CaMKII. Disruption of β_IV_-spectrin/CaMKII interaction or alanine substitution of β_IV_-spectrin Ser2250 (β_IV_-S2254A) prevented CaMKII-induced degradation, whereas a phosphomimetic construct (β_IV_-spectrin with glutamic acid substitution at serine 2254 [β_IV_-S2254E]) showed accelerated degradation in the absence of CaMKII. To assess the physiological significance of this phosphorylation event, we expressed exogenous β_IV_-S2254A and β_IV_-S2254E constructs in β_IV_-spectrin-deficient CFs, which have increased proliferation and fibrotic gene expression compared with WT CFs. β_IV_-S2254A but not β_IV_-S2254E normalized CF proliferation, gene expression, and contractility. Pathophysiological targeting of β_IV_-spectrin phosphorylation and subsequent degradation was identified in CFs activated with the profibrotic ligand angiotensin II, resulting in increased proliferation and signal transducer and activation of transcription 3 nuclear accumulation. While therapeutic delivery of exogenous WT β_IV_-spectrin partially reversed these trends, β_IV_-S2254A completely negated increased CF proliferation and signal transducer and activation of transcription 3 translocation. Moreover, we observed β_IV_-spectrin phosphorylation and associated loss in total protein within human heart tissue following heart failure. Together, these data illustrate a considerable role for the β_IV_-spectrin/CaMKII interaction in activating profibrotic signaling.

Increased fibrosis, characterized by excessive accumulation of extracellular matrix (ECM) protein, is a critical component of the repair and remodeling response to chronic neurohumoral or biomechanical stress in tissues throughout the body, including heart, lung, kidney, liver, skeletal muscle, and skin (1). In heart, fibrosis is not only important for replacement of damaged/dead myocardium in response to ischemic injury but also promotes electrical and mechanical dysfunction in a wide range of cardiac diseases (2, 3, 4). Cardiac fibroblasts (CFs) are the underlying cell types responsible for fibrotic remodeling. Under baseline (nondiseased) conditions, CFs play an essential supportive role in maintaining ECM homeostasis. Following injury, however, CFs undergo a transition from their basal state to an activated phenotype, characterized by enhanced proliferation, migration, and ECM deposition leading to increased fibrosis (5). Importantly, a diverse array of signaling events, including neurohumoral, biomechanical, and paracrine factors, has been identified in the CF activation process (3), providing opportunities for therapeutic strategies aimed at convergent molecular targets shared among these numerous signaling cascades.

Spectrins are a family of proteins initially identified for their role in providing structural support to cellular membranes (6, 7). The family includes two α- and five β-spectrin gene products where dimers of α- and β-spectrin interact with one another to form heterotetramers to facilitate interaction of membrane proteins with the actin cytoskeleton (8). Since their discovery, our understanding of spectrin function has grown to include critical spatiotemporal regulation of cell signaling events (9). Importantly, novel roles for the β_IV_-spectrin isoform have been discovered in organizing local signaling domains important for stress-induced cardiac remodeling, including arrhythmia and fibrosis (10, 11, 12, 13). Specifically, β_IV_-spectrin targets a subpopulation of the multifunctional Ca^2+/^calmodulin-dependent protein kinase II (CaMKII) to the cardiac myocyte intercalated disc membrane for regulation of voltage-gated Na^+^ channels. More recently, a broader role for β_IV_-spectrin/CaMKII has been identified in regulating gene programs in both cardiac myocytes and fibroblasts through interaction with the signal transducer and activation of transcription 3 (STAT3) (12, 13). Importantly, β_IV_-spectrin sequesters STAT3 near the membrane and out of the nucleus under basal (unstimulated) conditions. CaMKII activation in response to long-term pacing *in vitro* or chronic pressure overload *in vivo* promotes loss of β_IV_-spectrin and redistribution of STAT3 with nuclear accumulation, ultimately leading to changes in gene expression.

Here, we tested the hypothesis that β_IV_-spectrin is a novel target for direct CaMKII phosphorylation with effects on stability/expression of β_IV_-spectrin. Moreover, given the identified role for CaMKII in regulating cardiac fibrosis, we sought to explore involvement of the β_IV_-spectrin/CaMKII/STAT3 regulatory nexus in modulating CF gene expression and phenotypes. Using MS guided by a computational screen for consensus motifs, a putative CaMKII phosphorylation site was identified in the β_IV_-spectrin C terminus (Ser2250 in mouse and Ser2254 in human). Heterologous expression of human β_IV_-spectrin constructs lacking the putative site (β_IV_-spectrin with alanine substitution at serine 2254 [β_IV_-S2254A]) or mimicking constitutive phosphorylation (β_IV_-spectrin with glutamic acid substitution at serine 2254 [β_IV_-S2254E]) demonstrated a significance for this site in modulating the rate of β_IV_-spectrin degradation *in vitro*. Adenoviral expression of WT and mutant β_IV_-spectrin constructs in isolated mouse CFs further illustrated the importance for β_IV_-spectrin phosphorylation in regulating spectrin stability, STAT3 localization, gene expression, cell proliferation, and contractility in genetic models of spectrin deficiency and in response to angiotensin II (AngII). Finally, using a novel custom phospho-specific β_IV_-spectrin antibody, we identified increased β_IV_-spectrin phosphorylation at Ser2254 in human failing hearts, supporting an important role for this molecular pathway in human disease. These data advance our understanding of the dynamic range of β_IV_-spectrin function, including orchestration of its own degradation in response to chronic stress with coordinate changes in gene expression and cell function.

## Results

**Figure 1:**
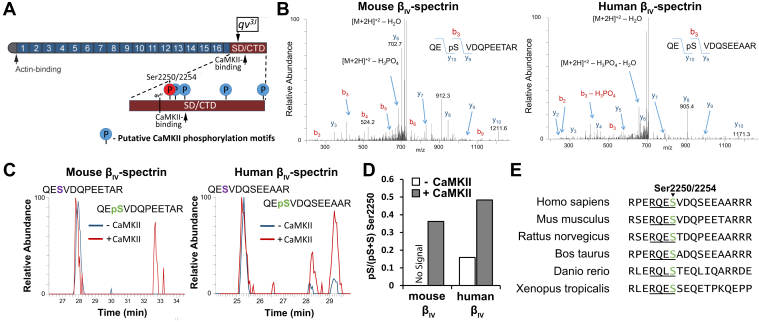
**Identification of CaMKII phosphorylation site in β**_**IV**_**-spectrin.***A*, schematic of β_IV_-spectrin protein domains. β_IV_-spectrin comprises an N-terminal actin-binding domain, 17 spectrin repeats, and C-terminal domain (CTD) and specific domain (SD). A computational screen identified five consensus phosphorylation motifs in the SD/CTC region of β_IV_-spectrin from both mouse and human using Group-Based Prediction System (GPS 3.0); serine residues 2250/2254 (highlighted in *red*), 2268/2272, 2301/2305, 2435/2438, and 2557/2560 for mouse and human, respectively. The *qv*^*3J*^ allele is a mutation resulting in a premature stop codon producing a truncated β_IV_-spectrin lacking the CaMKII binding motif as well as the putative phosphorylation sites. The *qv*^*4J*^ allele is a mutation resulting in a premature stop codon producing an extreme truncation ablating the STAT3-binding residue. *B*, the MS/MS spectra for mouse and human β_IV_-spectrin phosphopeptides, (2248)QEpSVDQPEETAR(2259) [mouse] and (2252)QEpSVDQSEEAAR(2263) [human] are shown. COS-7 cells expressing mouse and human HA-tagged β_IV_-spectrin ± constitutively active CaMKII (T287D) were used. The mouse doubly charged peptide has an observed *m*/*z* of 714.7884 Da. These spectra contain H_3_PO_4_ loss from the precursor, which is consistent with the presence of a phosphorylated serine residue, whereas the mass difference between the y10 and y9 ions is consistent with phosphorylation at S2250. The human doubly charged peptide has an observed *m*/*z* of 734.8034 Da. The mass difference between the y10 and y9 ions is consistent with phosphorylation at S2254. *C*, chromatograms were plotted for the unmodified and phosphorylated forms of the mouse and human peptides for both the minus and plus CaMKII samples. The degree of pS2250/2254 increases in the presence of CaMKII. *D*, integrated areas from chromatograms showing increase in phosphorylated peptide in the presence of CaMKII. Data reflect percentages from a single replicate for each group. *E*, alignment of β_IV_-spectrin protein sequence showing conservation across species of the putative CaMKII target sequence (*underlined*) and the phosphorylated serine (*green*). CaMKII, Ca^2+^/calmodulin-dependent kinase II; CTC, C-terminal construct; HA, hemagglutinin; MS/MS, tandem MS/MS; *qv*^*3J*^, mutant β_IV_-spectrin allele C-terminal region containing CaMKII interaction motif; *qv*^*4J*^, mutant β_IV_-spectrin allele lacking repeats 10 through C terminus; STAT3, signal transducer and activation of transcription 3.

Previous results revealed that chronic pressure overload induced prominent loss of β_IV_-spectrin in WT mice. However, mutant mice expressing truncated β_IV_-spectrin lacking an identified CaMKII interaction motif (mutant β_IV_-spectrin allele C-terminal region containing CaMKII interaction motif [*qv*^*3J*^] allele) resulted in maintained β_IV_-spectrin protein expression (12). Furthermore, *in vitro* studies showed that rapid pacing of WT myocytes under hyperphosphorylating conditions recapitulated loss of β_IV_-spectrin observed *in vivo*, which was prevented by the CaMKII inhibitor autocamtide-2-related inhibitory peptide as well as when using *qv*^*3J*^ myocytes (12). Based on these data, we hypothesized that CaMKII directly phosphorylates β_IV_-spectrin to induce degradation in response to stress. Initial bioinformatics screening of the fragment absent in the *qv*^*3J*^ β_IV_-spectrin allele revealed several putative CaMKII phosphorylation motifs found in both mouse and human, including serine residues 2250, 2268, 2301, 2435, and 2557 (2254, 2272, 2305, 2438, and 2560 in human) (Fig. 1*A*). Notably, Ser2250/2254 received the highest score as a predicted phosphorylation target (residue highlighted in *red*, Fig. 1*A*). We next performed MS analysis on immunoprecipitated β_IV_-spectrin derived from COS-7 cell lysates transfected with hemagglutinin (HA)-tagged versions of mouse or human β_IV_-spectrin encoding spectrin repeats 10 through the C terminus. These constructs were coexpressed with or without constitutively active CaMKII. Phosphorylated and nonphosphorylated tryptic peptide fragments were identified corresponding to β_IV_-spectrin residues 2247 to 2259 in mouse (mβ_IV,2247–2259_) and residues 2251 to 2263 in human (hβ_IV,2251–2263_) (associated tandem MS/MS [MS/MS] spectra are shown in Fig. 1*B,* Table S1). The percentage of phosphorylated mβ_IV,2247–2259_ (out of total detected mβ_IV,2247–2259_) increased from 0% in the absence of CaMKII to 36.2% when coexpressed with CaMKII. A similar effect was observed for the human sequence with an increase in phosphorylated hβ_IV,2251–2263_ from 15.9% to 48.3% in the absence and presence of CaMKII, respectively (Fig. 1*D*). Notably, human β_IV_-spectrin achieved higher percentages of phosphorylated peptide at both baseline and in response to constitutively active CaMKII coexpression, suggesting that differences in steady-state levels of phosphorylation may exist between mouse and human β_IV_-spectrin. Importantly, the identified fragment and corresponding phosphorylation site (Ser2250 in the mouse and Ser2254 in human) are highly conserved across species (Fig. 1*E*), supporting the physiological significance. MS/MS spectra were unable to detect peptide fragments for the other remaining predicted CaMKII target motifs, preventing assessment of potential phosphorylation of additional sites.

**Figure 2:**
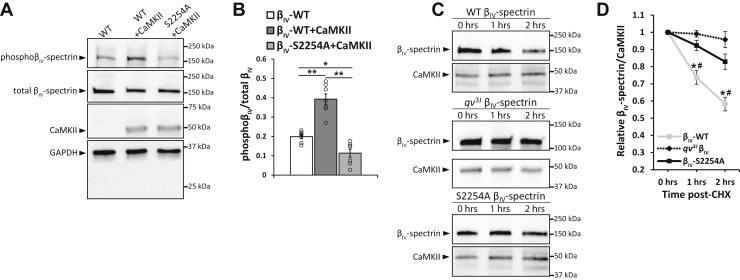
**CaMKII-dependent phosphorylation of β**_**IV**_**-spectrin at S2254 promotes degradation.***A*, representative immunoblots and *B*, densitometric measurements for phospho-β_IV_-spectrin (S2254) and total β_IV_-spectrin from COS-7 cells transfected for 48 h with WT β_IV_-spectrin ± CaMKII T287D or phosphoablated β_IV_-spectrin (S2254A) with CaMKII T287D. ∗*p* < 0.05 and ∗∗*p* < 0.01 by one-way ANOVA followed by Tukey HSD test (*F* = 42.67; *p* < 0.0001); n = 7. *C*, representative immunoblots (bands at expected size for expressed constructs comprising repeat 10 through the spectrin C terminus) and *D*, densitometric measurements from COS-7 cells transfected with WT, *qv*^*3J*^, or S2254A β_IV_-spectrin coexpressed with constitutively active CaMKII, T287D. Cells were cultured for 48 h after which degradation assays were performed by treating COS-7 cells with CHX (20 μM) to stop new protein synthesis for 1 and 2 h to measure the rate of β_IV-_spectrin loss. β_IV_-spectrin expression was normalized against cotransfected CaMKII to account for transfection control. ∗*p* < 0.05 *versus* WT and #*p* < 0.05 *versus qv*^*3J*^ by one-way ANOVA followed by Tukey HSD test (*F* = 14.68, *p* = 0.0006, 1 h; *F* = 15.33, *p* = 0.0005, 2 h); n = 5. Summary data are presented as mean ± SEM. CaMKII, Ca^2+^/calmodulin-dependent kinase II; CHX, cycloheximide; HSD, honest significant difference; *qv*^*3J*^, mutant β^IV^-spectrin allele C-terminal region containing CaMKII interaction motif.

Based on our MS results, we generated a rabbit affinity-purified polyclonal antibody for detection of phosphorylated β_IV_-spectrin Ser2250/2254. The antibody was tested by immunoblot on lysates generated from COS-7 cells transfected with human β_IV_-spectrin encoding repeats 10 through the C terminus (WT β_IV_-spectrin) alone, WT β_IV_-spectrin with constitutively active CaMKII, or a phosphoablated β_IV_-spectrin derived from the WT β_IV_-spectrin construct utilizing a serine to alanine mutation at residue 2254 (β_IV_-S2254A) and coexpressed with constitutively active CaMKII. Increased immunoreactive signal was observed with CaMKII coexpressed with WT β_IV_-spectrin but not β_IV_-S2254A (Fig. 2, *A* and *B*). In order to more thoroughly evaluate antibody specificity, preadsorption with the antigenic peptide used in antibody generation and affinity purification was performed. As expected, this resulted in the complete loss of signal when treated with the phosphorylated peptide (Fig. S1*A*). Further antibody characterization was performed by pretreatment of the membrane with alkaline phosphatase to eliminate immunoreactive signal corresponding to phosphorylation of β_IV_-spectrin. This resulted in signal loss for both WT β_IV_-spectrin alone (identified to have basal phosphorylation status in MS data; Fig. 1*A*) and WT β_IV_-spectrin with CaMKII but marginal loss for β_IV_-S2254A with CaMKII (Fig. S1, *B* and *C*). It is important to note that all validation experiments indicate some degree of crossreactivity with unphosphorylated epitopes. Treatment with the unmodified antigenic peptide also resulted in partial reduction of signal for all evaluated conditions, suggesting partial reactivity with unphosphorylated β_IV_-spectrin protein. Also, residual immunoreactive signal was apparent in cells expressing β_IV_-S2254A + CaMKII and in samples pretreated with alkaline phosphatase. Together, these data support the importance of including appropriate controls (*i.e.*, normalization to total β_IV_-spectrin immunoreactive signal) when using the antibody. That said, these data support the ability of the antibody to detect phosphorylation of β_IV_-spectrin at S2254.

**Figure 3:**
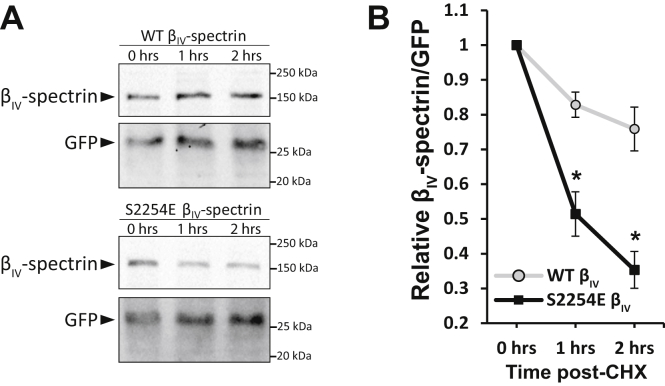
**Constitutive phosphorylation of β**_**IV**_**-spectrin at S2254 induces degradation.***A*, representative immunoblots and *B*, associated summary data from COS-7 cells transfected with WT β_IV_-spectrin or phosphomimetic β_IV_-spectrin (S2254E) and coexpressed with GFP. Degradation assays were performed by treating COS-7 cells with CHX (20 μM) to stop new protein synthesis for 1- and 2-h time points to measure the rate of β_IV_-spectrin loss. β_IV_-spectrin expression was normalized against cotransfected GFP to account for transfection control. Resulting values normalized to baseline. ∗*p* < 0.05 *versus* WT by Student's two-tailed *t* test; n = 3. Summary data are presented as mean ± SEM. CHX, cycloheximide.

We next tested the hypothesis that CaMKII-dependent phosphorylation of β_IV_-spectrin modulates spectrin stability/expression. We cotransfected COS-7 cells with constitutively active CaMKII and the WT and β_IV_-S2254A constructs. We also included a *qv*^*3J*^ construct lacking the C-terminal region containing CaMKII binding and phosphorylation site (starting at spectrin repeat 10 as the case for WT and β_IV_-S2254A). Cells were treated with cycloheximide (CHX) 48 h post-transfection to stop new protein synthesis, allowing for assessment of potential differences in protein degradation. Lysates were collected at baseline, as well as 1 and 2 h post-CHX treatment to evaluate the rate of β_IV_-spectrin loss. WT β_IV_-spectrin levels (expressed as percentage of baseline expression) were significantly lower 2 h post-CHX treatment compared with *qv*^*3J*^ or β_IV_-S2254A (Fig. 2, *C* and *D*), without dramatic changes at longer time points (up to 24 h, data not shown). Notably, there was no difference in relative β_IV_-S2254A and *qv*^*3J*^ expression at 2 h post-CHX. Given that MS results were unable to detect coverage of the other predicted phosphorylation sites (S2272, S2305, S2438, and S2560), a series of phosphoablated mutants were generated to similarly test their functional significance in regulating β_IV_-spectrin loss. Coexpression of these additional mutants with constitutively active CaMKII led to no change in the rate of β_IV_-spectrin loss compared with WT (Fig. S2), reinforcing a central role for Ser2254.To subsequently determine whether phosphorylation of Ser2254 was sufficient to induce spectrin degradation, COS-7 cells were transfected with phoshomimetic β_IV_-spectrin (β_IV_-S2254E) or WT constructs in the absence of constitutively active CaMKII (GFP cotransfected with β_IV_-spectrin instead as the cotransfected control). Following treatment with CHX, we again assessed levels of β_IV_-spectrin expression, which identified a significant loss of spectrin with the β_IV_-S2254E but not WT construct, even in the absence of CaMKII coexpression (Fig. 3, *A* and *B*). Together, these data support an important role for Ser2254 phosphorylation in modulating stability/expression of β_IV_-spectrin.

**Figure 4:**
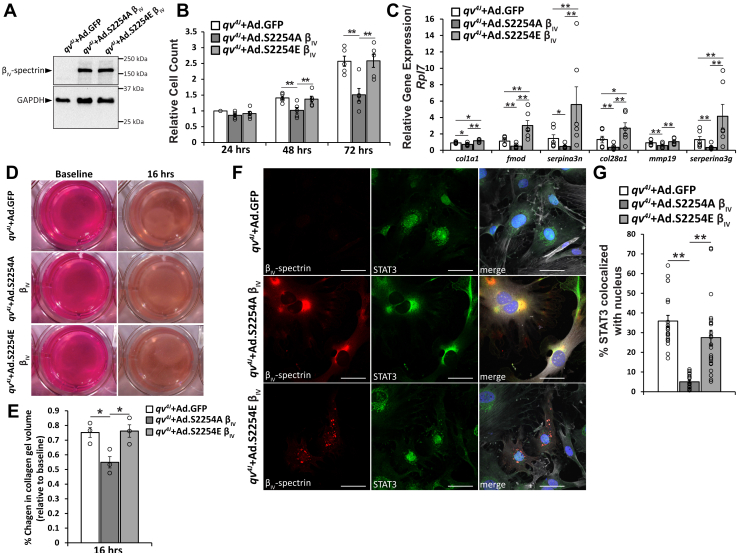
**CaMKII-dependent phosphorylation of β**_**IV**_**-spectrin alters cardiac fibroblast (CF) gene expression and function.***A*, representative immunoblots of CF lysates from *qv*^*4J*^ mice expressing truncated β_IV_-spectrin lacking interaction with the transcription factor STAT3. CFs were transduced with adenovirus-encoding GFP (control, Ad.GFP), human phosphoablated S2254A β_IV_ (Ad.β_IV_-S2254A), or human phosphomimetic S2254E β_IV_ (Ad.β_IV_-S2254E), validating functional and comparable level of expression from adenovirus delivery. *B*, summary data of manual cell counts from *qv*^*4J*^ CFs expressing Ad.GFP (control), Ad.β_IV_-S2254A, and Ad.β_IV_-S2254E. Cell counts were taken at 24, 48, and 72 h post-transduction. Data are normalized to 24 h control condition. ∗∗*p* < 0.01 by correlated one-way ANOVA followed by Tukey HSD test (*F* = 15.51, *p* = 0.0018, 48 h; *F* = 29.25, *p* = 0.0002, 72 h); n = 5. *C*, summary data for expression of select profibrotic genes (relative to *Rpl7*) evaluated by quantitative PCR from *qv*^*4J*^ CFs 72 h after transduction with Ad.GFP, Ad.β_IV_-S2254A, or Ad.β_IV_-S2254E. ∗*p* < 0.05 and ∗∗*p* < 0.01 by correlated one-way ANOVA followed by Tukey HSD test (*F* = 22.76, *p* = 0.0002, *col1a1*; *F* = 49.22, *p* < 0.0001, *fmod*; *F* = 22.64, *p* = 0.0002, *serpina3n*; *F* = 34.03, *p* < 0.0001, *col28a1*; *F* = 23.43, *p* = 0.0002, *mmp19*; and *F* = 55.23, *p* < 0.0001, *serpina3g*); n = 6. *D*, representative images of collagen gels seeded with *qv*^*4J*^ CFs expressing Ad.GFP, Ad.β_IV_-S2254A, or Ad.β_IV_-S2254E just after collagen gel formation and release (baseline) and 16 h after incubation. *E*, summary data of the change in collagen gel volume over the course of 16 h in Ad.GFP, Ad.β_IV_-S2254A, or Ad.β_IV_-S2254E conditions. ∗*p* < 0.05 by one-way ANOVA followed by Tukey HSD test (*F* = 6.51; *p* = 0.0314); n = 3. *F*, representative confocal microscopy images (60× magnification) of permeabilized *qv*^*4J*^ CFs 48 h post-transduction with Ad.GFP, Ad.β_IV_-S2254A, or Ad.β_IV_-S2254E immunostained for β_IV_-spectrin (*red*), STAT3 (*green*), phalloidin (*gray* in merged image), and DAPI (*blue* in merged image). The scale bar represents 50 μm. *G*, summary data of STAT3 nuclear localization in Ad.GFP-, Ad.β_IV_-S2254A-, or Ad.β_IV_-S2254E-treated *qv*^*4J*^ CFs. Data were analyzed from at least five different fields per preparation. ∗∗*p* < 0.01 by one-way ANOVA followed by Tukey HSD test (*F* = 29.76; *p* < 0.0001); n = 4 experiments from three different biologic preparations. Summary data are presented as mean ± SEM. CaMKII, Ca^2+^/calmodulin-dependent kinase II; DAPI, 4′,6-diamidino-2-phenylindole; HSD, honest significant difference; STAT3, signal transducer and activation of transcription 3; *qv*^*4J*^, mutant β^IV^-spectrin allele lacking repeats 10 through C terminus.

CFs isolated from β_IV_-spectrin–deficient mice (mutant β_IV_-spectrin allele lacking repeats 10 through C terminus [*qv*^*4J*^] mice expressing truncated β_IV_-spectrin lacking repeats 10 through the C terminus) were previously shown to have enhanced expression of profibrotic genes, increased proliferation, increased contractility, and associated *in vivo* fibrosis compared with WT animals (13). Therefore, as a first step in assessing the potential physiological significance of β_IV_-spectrin phosphorylation at S2254, we evaluated CF phenotype in *qv*^*4J*^ CFs, as a model for β_IV_-spectrin deficiency. *qv*^*4J*^ CFs were subjected to adenoviral expression of human phosphoablated (Ad.β_IV_-S2254A) or phosphomimetic (Ad.β_IV_-S2254E) β_IV_-spectrin or control (Ad.GFP) (Fig. 4*A*). Mouse and human β_IV_-spectrin share 96% sequence homology, supporting use of human constructs in mouse CFs. Importantly, *qv*^*4J*^ CFs expressing Ad.β_IV_-S2254A showed a significant reduction in proliferation at both 48 and 72 h post-transduction compared with Ad.β_IV_-S2254E or control (Fig. 4*B*). Furthermore, delivery of Ad.β_IV_-S2254A significantly reduced CF contractility in relation to Ad.β_IV_-S2254E or Ad.GFP treated cells, as assessed by collagen gel volume compaction (Fig. 4, *D* and *E*) as another functional readout for profibrotic activity. At the same time, a significant reduction in select profibrotic genes was observed in Ad.β_IV_-S2254A expressing CFs compared with Ad.β_IV_-S2254E or control (Fig. 4*C*). Interestingly, while CFs expressing Ad.β_IV_-S2254E showed a similar proliferation rate compared with control, they had significant enhancement of profibrotic gene expression. This change in fibrotic gene expression was specific to the recognized β_IV_-spectrin/STAT3 pathway, as evaluation of additional fibrosis-associated genes (*pdgfra*, *postn*, and *vim*) previously shown to be unaffected by β_IV_-spectrin/STAT3 (13) remained unregulated between groups (Fig. S3).

To ensure that observed phenotypic changes with expression of β_IV_-spectrin constructs were not an artifact of the *qv*^*4J*^ model (expression of truncated β_IV_-spectrin), we repeated a subset of experiments using our fibroblast-specific β_IV_-spectrin model utilizing a periostin^MerCreMer^ crossed with β_IV_-spectrin–floxed mice (inducible fibroblast-specific β_IV_-spectrin knockout mouse [β_IV_ifKO]) (Fig. S4*A*) (13). Periostin expression is highly specific to activated CFs and can be induced with AngII. Therefore, β_IV_ifKO and control (β_IV_-floxed, Cre−) mice were treated for 7 days with tamoxifen/AngII for activation of the MerCreMer periostin promoter to induce β_IV_-spectrin KO in CFs (Fig. S4, *A* and *B*), as previously described (13). CFs isolated from β_IV_ifKO mice were then transduced with Ad.β_IV_-S2254A, Ad.β_IV_-S2254E, or Ad.GFP. Consistent with the phenotype observed in the *qv*^*4J*^-derived CFs, Ad.β_IV_-S2254A treatment reduced contractility (as assessed by collagen gel compaction) compared with Ad.β_IV_-S2254E and Ad.GFP treated cells. Collectively, these data support a functional role for the phosphorylation of β_IV_-spectrin at S2254 in its ability to regulate CF gene expression and profibrotic activity.

β_IV_-spectrin alters CF gene expression by controlling subcellular localization of the transcription factor STAT3 through physical interaction and sequestration at the membrane (12, 13). Notably, conditions of β_IV_-spectrin loss through genetic or acquired means untethers STAT3, resulting in greater nuclear expression and transcriptional activity. To determine whether phosphorylation of β_IV_-spectrin at S2254, and subsequent changes in β_IV_-spectrin stability, altered STAT3 localization, immunostaining was performed on *qv*^*4J*^ CFs expressing Ad.β_IV_-S2254A, Ad.β_IV_-S2254E, or Ad.GFP. Consistent with previous observations, control (Ad.GFP) *qv*^*4J*^ CFs displayed prominent nuclear localization of STAT3 because of the absence of STAT3/β_IV_-spectrin binding in the *qv*^*4J*^ allele (Fig. 4, *F* and *G*). Expression of Ad.β_IV_-S2254A but not Ad.β_IV_-S2254E in *qv*^*4J*^ CFs significantly reduced the relative amount of STAT3 in the nucleus compared with control, consistent with proliferation, gene expression, and compaction assay results. Interestingly, Ad.β_IV_-S2254A was colocalized with STAT3 in a diffuse and perinuclear pattern, whereas Ad.β_IV_-S2254E showed a distinct punctate pattern (Fig. 4*F*).

**Figure 5:**
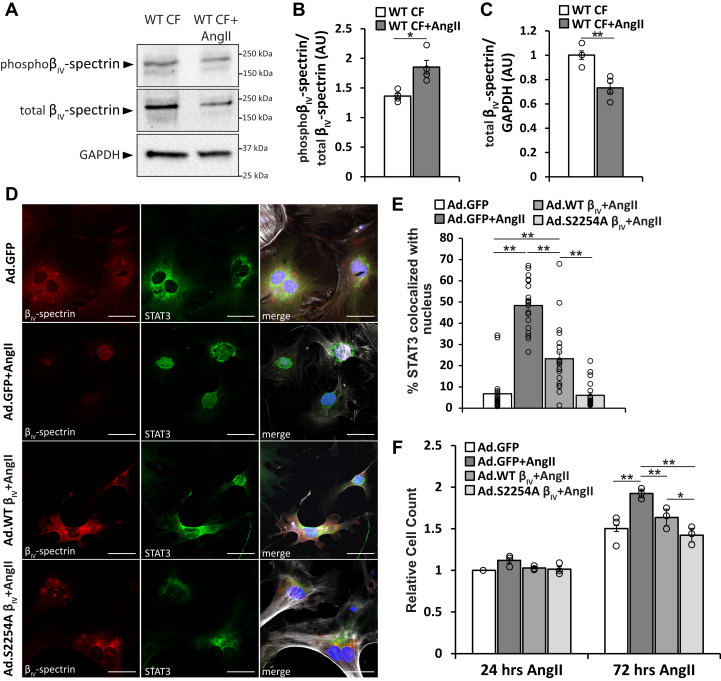
**Angiotensin II (AngII) induces β**_**IV**_**-spectrin phosphorylation.***A*, representative immunoblots for phospho-β_IV_-spectrin (S2250) and total β_IV_-spectrin in WT mouse cardiac fibroblasts (CFs) ± AngII (100 nM) for 48 h. *B*, associated densitometric measurements for phospho-β_IV_-spectrin normalized to total β_IV_-spectrin and *C*, total β_IV_-spectrin normalized to GAPDH. ∗*p* < 0.05 and ∗∗*p* < 0.01 by Student's two-tailed *t* test; n = 4. *D*, representative confocal microscopy images (60× magnification) of permeabilized WT CFs following 48 h transduction of adenovirus-encoding GFP (Ad.GFP) at baseline (no AngII) or Ad.GFP, Ad.β_IV_-WT, or Ad.β_IV_-S2254A and 24 h treatment with AngII (100 nM) immunostained for β_IV_-spectrin (*red*), STAT3 (*green*), phalloidin (*gray* in merged image), and DAPI (*blue* in merged image). The scale bar represents 50 μm. *E*, summary data of STAT3 nuclear localization in WT CFs expressing Ad.GFP ± AngII, Ad.β_IV_-WT + AngII, or Ad.β_IV_-S2254A + AngII. Data were analyzed from at least five different fields per preparation. ∗∗*p* < 0.01 by one-way ANOVA followed by Tukey HSD test (*F* = 65.49; *p* < 0.0001); n = 4 experiments from three different biologic preparations. *F*, summary data of manual cell counts from WT CF with the same treatment conditions taken at 24 and 72 h after AngII treatment. ∗*p* < 0.05 and ∗∗*p* < 0.01 by correlated one-way ANOVA followed by Tukey HSD test (*F* = 32.28; *p* = 0.0004); n = 3. Summary data are presented as mean ± SEM. DAPI, 4′,6-diamidino-2-phenylindole; HSD, honest significant difference; STAT3, signal transducer and activation of transcription 3.

Beyond genetic models of β_IV_-spectrin deficiency (*qv*^*4J*^ and β_IV_ifKO), we sought to test whether β_IV_-spectrin phosphorylation at Ser2250/2254 influenced CF phenotype under pathophysiological stress conditions involving AngII treatment, which alters CF phenotype (including increased proliferation) in part through activation of CaMKII (14, 15, 16, 17, 18). CFs treated with AngII (1 μM) showed an increase in phosphorylated β_IV_-spectrin (expressed as fraction of total) with an overall decrease in total β_IV_-spectrin expression (Fig. 5, *A*–*C*). To determine whether phosphorylation of β_IV_-spectrin regulated the CF functional response to AngII, WT CFs were transduced with Ad.β_IV_-WT, Ad.β_IV_-S2254A, or Ad.GFP prior to treatment with AngII. Consistent with immunoblot data, AngII treatment led to a loss in β_IV_-spectrin signal in Ad.GFP-expressing CFs (Fig. 5*D*), together with a significant increase in STAT3 nuclear signal and increased proliferation compared with Ad.GFP-expressing CFs (Fig. 5, *D*–*G*). Expression of Ad.β_IV_-WT significantly reduced AngII-induced translocation of STAT3 into the nucleus and reduced the proliferation rate compared with Ad.GFP-expressing CFs. Interestingly, Ad.β_IV_-S2254A-expressing CFs were resistant to AngII-induced STAT3 nuclear accumulation with levels comparable to untreated Ad.GFP-expressing CFs (Fig. 5, *D* and *F*). Similarly, Ad.β_IV_-S2254A-expressing CFs treated with AngII showed a similar proliferation rate to untreated Ad.GFP CFs. Together, these studies establish a link between β_IV_-spectrin phosphorylation, STAT3 localization, and CF profibrotic phenotype.

**Figure 6:**
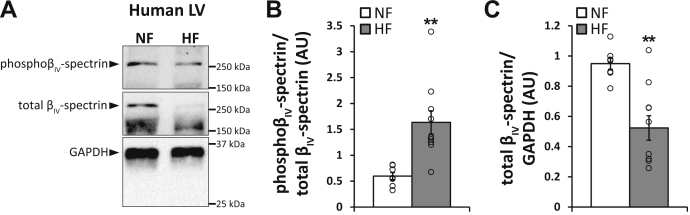
**Increased β**_**IV**_**-spectrin phosphorylation in human failing hearts.***A*, representative immunoblots for phospho-β_IV_-spectrin (S2254) and total β_IV_-spectrin in human left ventricular nonfailing (NF) and heart failure (HF) tissue samples. *B*, associated summary data for phospho-β_IV_-spectrin normalized to total β_IV_-spectrin and *C*, total β_IV_-spectrin normalized to GAPDH. HF samples comprise both ischemic and nonischemic samples (groups combined). ∗*p* < 0.05 and ∗∗*p* < 0.01 by Student's two-tailed *t* test; NF: n = 7, HF: n = 10. Summary data are presented as mean ± SEM.

Finally, as a first step in determining whether our findings in mouse translate to human, levels of phosphorylated β_IV_-spectrin were evaluated in ventricular lysates from normal (nonfailing) and failing human hearts. A significant increase in phosphorylated β_IV_-spectrin (as a fraction of total β_IV_-spectrin) was observed together with a decrease in overall β_IV_-spectrin levels in heart failure–compared control (Fig. 6, *A*–*C*), suggesting a potential role for this regulatory site in human disease.

## Discussion

Here, we sought to identify the molecular mechanism for CaMKII-dependent regulation of β_IV_-spectrin expression and CF activity. Using a computational screen, MS analysis, and *in vitro* functional assays, we identified a putative CaMKII phosphorylation site in the β_IV_-spectrin C terminus conserved across species, including mouse and human (S2250/S2254). We observed that ablation of the CaMKII phosphorylation site in β_IV_-spectrin (β_IV_-S2254A) conferred resistance to CaMKII-induced degradation *in vitro*, whereas a phosphomimetic construct (β_IV_-S2254E) demonstrated accelerated loss even in the absence of CaMKII. Using a custom antibody, we observed that AngII treatment of isolated mouse CFs induced phosphorylation of β_IV_-spectrin together with increased nuclear localization of STAT3, increased proliferation, and contractility. Adenoviral expression of WT β_IV_-spectrin was able to partially prevent AngII-induced changes in STAT3 localization and proliferation in CFs. Moreover, CFs expressing Ad.β_IV_-S2254A treated with AngII were almost indistinguishable from untreated control with respect to STAT3 localization and CF phenotype. Finally, we reported an increase in phosphorylated β_IV_-spectrin in samples from human failing hearts compared with nonfailing controls, suggesting a significant role for β_IV_-spectrin phosphorylation and subsequent protein loss in pathologic cardiac remodeling. Our findings expand the dynamic nature of β_IV_-spectrin to include coordination of its own disassembly in the presence of chronic stress. Furthermore, the system is imbued with a mechanism for reporting its fate back to the cell through STAT3-dependent changes in gene expression.

Recent studies have identified a role for β_IV_-spectrin in mediating the fibrotic response in heart. Both cardiomyocyte- and CF-specific deletion of β_IV_-spectrin led to a prominent increase in fibrosis, arrhythmia, and contractile dysfunction (12, 13). Conversely, disruption of β_IV_-spectrin/CaMKII interaction (*qv*^*3J*^ allele) preserved β_IV_-spectrin expression, abrogated fibrosis, and improved cardiac function in response to pressure overload. Together, these studies intimately tie β_IV_-spectrin expression with the onset of pathologic remodeling through both genetic and acquired disease pathways. Our new findings reveal the mechanistic relationship between β_IV_-spectrin stability and CaMKII *via* direct phosphorylation of a conserved residue in the β_IV_-spectrin C terminus. Although the phosphorylation site identified here is distinct from and proximal to (44 amino acids upstream) the previously identified CaMKII-binding site in β_IV_-spectrin (give residues), these studies cannot rule out the possibility that CaMKII phosphorylation alters CaMKII binding to β_IV_-spectrin itself with potential disruptions in associated signaling (10). While studies here concentrate on implications of β_IV_-spectrin phosphorylation/expression related to STAT3 signaling, it will be interesting in the future to assess consequences for CaMKII signaling.

β_IV_-spectrin expression is closely linked to that of α_II_-spectrin, which associate together in heterotetrameric complexes. Indeed, genetic deletion of α_II_-spectrin was found to promote marked reductions in β_IV_-spectrin (19), suggesting a destabilizing effect by the loss of interacting partners. Interestingly, α_II_-spectrin also experiences protein loss in heart failure, whereas mouse models of cardiac-specific deletion of α_II_-spectrin increases fibrosis at baseline and disease states (20), reflecting states of remodeling consistent with β_IV_-spectrin loss. Despite this, it is not clear what role α_II_-spectrin might play in the CaMKII-dependent degradation of β_IV_-spectrin and subsequent fibrotic remodeling. Is α_II_-spectrin stability simply dependent on signaling events targeting β_IV_-spectrin, or might there exist independent regulation of α_II_-spectrin that could dictate outcomes for β_IV_-spectrin expression and fibrosis? Overall, this codependent regulation may even suggest that multiple disease pathways are able to converge on this spectrin complex to facilitate activation of common underlying remodeling events.

The identification of β_IV_-spectrin as a novel target for CaMKII adds to a growing list of CaMKII substrates involved in disease remodeling (21, 22, 23, 24). Importantly, CaMKII has long been distinguished as a critical factor in mediating pathologic electrical remodeling, hypertrophic signaling, and fibrosis (22, 25, 26). Genetic models of CaMKII overexpression result in spontaneous hypertrophy, decreased cardiac function, and increased mortality (27, 28, 29). In contrast, genetic and pharmacologic inhibition of CaMKII provides protection from chronic stress, limiting the development of cardiomyopathy and maintaining cardiac output in both ischemic and nonischemic disease models (30, 31, 32, 33). Functional effects of CaMKII activity are mediated through post-translational modifications to a range of ion-handling proteins such as phospholamban (34), ryanodine receptor (35), and the voltage-gated channels Ca_v_1.2 (36) and Na_v_1.5 (10, 37), as well as more direct hypertrophic drivers like the class 2 histone deacetylases 4 and 5 (38). Notably, our previous investigations found that the β_IV_-spectrin *qv*^*3J*^ mouse with disruption of CaMKII/β_IV_-spectrin interaction led to reduced fibrosis but maintained a hypertrophic response to chronic pressure overload (12), delineating the multiple etiologies of CaMKII-directed remodeling and the pivotal importance of β_IV_-spectrin stability with regard to fibrosis. Indeed, direct inhibition of CaMKII in CFs has been shown to reduce CF activation and fibrosis (17, 18), further reinforcing the role for a CaMKII/β_IV_-spectrin axis of regulation.

CaMKII resides at the hub of a vast signaling network and is well equipped to integrate a host of pathophysiological signaling inputs. Neurohumoral signaling, ischemic stress, inflammation, reactive oxygen species, and diabetes are all associated with activation of CaMKII resulting in cardiomyopathy and fibrosis (30, 39, 40), whereas specific profibrotic cytokines and neurohumoral factors such as AngII (41), interleukin-6 (42), tumor necrosis factor beta (17), and bone morphogenetic protein 4 (43) also provide CaMKII activation. Based on our findings that CaMKII-dependent phosphorylation of β_IV_-spectrin modulates gene expression and activity of CFs at baseline and in response to AngII, it is interesting to consider the possibility that the CaMKII/β_IV_-spectrin axis exists as a central common pathway for fibrotic remodeling response to stress.

Our data support that the CaMKII/β_IV_-spectrin signaling node resides upstream of the transcription factor STAT3, whereas loss of β_IV_-spectrin has been shown to drive fibrotic remodeling by promoting redistribution of STAT3 into the nucleus and subsequent changes in profibrotic genes (12, 13). These data add to the emerging status of STAT3 as a master controller of the fibrotic response (44). Pharmacologic inhibition of STAT3 directly (12, 45, 46) or genetic ablation of upstream STAT3 activators, such as interleukin-6 (42), have successfully attenuated cardiac fibrosis in both the atria and/or ventricles following ischemia and pressure overload, reducing adverse electrical remodeling and improving heart function. Moreover, STAT3 activation and nuclear translocation has been shown to be upstream of CaMKII activity and can be impaired by direct CaMKII inhibition (42). Importantly, the investigation here is not only supportive of these data but identifies β_IV_-spectrin as the bridge between these signaling events, showing that stable expression of phosphoablated β_IV_-spectrin is able to reduce nuclear STAT3 and CF proliferation even in the presence of CaMKII activation.

Characteristically, CaMKII activity often leads to altered function for its target substrate; however, we describe here the unexpected consequence of induced protein degradation. While there is precedence for phosphorylation-dependent degradation of target substrates for other protein kinases (47, 48, 49), such a mechanistic role for CaMKII is less established. Up to now, just one such target has been identified in the protein Liprinα1, a regulator for neuronal dendrite maturation (50). Interestingly, CaMKII phosphorylation of Liprinα1 activates a PEST sequence in the target protein, characterized by the enrichment of proline, glutamate, serine, and threonine residues. Such sequences facilitate basal regulation of protein half-lives as well as induced degradation following post-translational modification, recruiting both calpain and proteosome-dependent degradation pathways. It will be interesting to further analyze the β_IV_-spectrin C terminus for putative PEST sequences to determine whether a similar mechanism may be involved in the behavior observed here. Importantly, other clues to the mechanisms responsible for CaMKII-dependent spectrin loss may be found in the confocal imaging studies of β_IV_-spectrin localization. Specifically, phosphomimetic and WT β_IV_-spectrin stimulated with AngII showed significant punctate patterns of localization (Figs. 4*F* and 5*D*), consistent with those for both proteosomal complexes and/or lysosomal bodies (51, 52). Notably, these puncta were absent when expressing β_IV_-spectrin under nondegrading conditions such as phosphoablated β_IV_-spectrin with AngII (Fig. 5*D*), highlighting a clear association with Ser2254 phosphorylation status, β_IV_-spectrin stability, and nuclear STAT3 accumulation.

Overall, this study identifies β_IV_-spectrin as a novel target of CaMKII and reveals CaMKII/β_IV_-spectrin signaling to play an important role in modulating the response of CFs to neurohumoral stress. Given the paramount role that CaMKII activation plays in multiple cardiac disease pathways and the established significance for β_IV_-spectrin in regulating cardiac fibrosis, the β_IV_-spectrin/CaMKII axis represents a comprehensive event and attractive therapeutic candidate in fibrotic remodeling.

## Experimental procedures

### Mouse models

Adult (2–4 months) C57BL/6J male and female WT and truncated β_IV_-spectrin (*qv*^*4J*^) littermate mice were used. The *qv*^*4J*^ mice were obtained from Jackson Laboratory and express a *Sptnb4* allele with a spontaneous insertion point mutation at C4234T (Q1358 → Stop) resulting in a premature stop codon proximal to the STAT3-binding region in β_IV_-spectrin (53, 54). Periostin^MerCreMer^ mice (55) were crossbred with β_IV_-spectrin–floxed mice (12) to obtain tamoxifen-inducible β_IV_-ifKO mice. Adult male and female β_IV_-ifKO or control (β_IV_-spectrin floxed, Cre−) mice were treated with tamoxifen (MilliporeSigma) dissolved in corn oil (75 mg/kg i.p. injection daily) and AngII (2.16 mg/kg i.p. injection daily) for 1 week to induce MerCreCre expression under regulation of the periostin promoter (56, 57). Animal studies were conducted in accordance with the Guide for the Care and Use of Laboratory Animals published by the National Institutes of Health (NIH) following protocols that were reviewed and approved by the Institutional Animal Care and Use Committee at The Ohio State University.

### Bioinformatics screening of putative CaMKII phosphorylation target sites in β_IV_-spectrin

A C-terminal fragment of the β_IV_-spectrin protein was evaluated for potential CaMKII target phosphorylation motifs using the Group-Based Prediction System (GPS 3.0) (58). Five potential sites of interest were identified that were conserved between mouse and human β_IV_-spectrin sequences (residues refer to position in human sequence): Ser2254, Ser2272, Ser2305, Ser2438, and Ser2560.

### MS

A fragment of β_IV_-spectrin from spectrin repeat 10 through the C terminus (representing a natively expressed truncated version of β_IV_-spectrin) was cloned from mouse and human complementary DNA (cDNA) libraries into pcDNA3.1 with an HA tag for heterologous expression, as previously described (12). HA-tagged β_IV_-spectrin was expressed in COS-7 cells (American Type Culture Collection catalog no. CRL-1651, RRID: CVCL 0224) and purified using Anti-HA antibody (3724; Cell Signaling) loaded onto TrueBlot agarose beads (00-8800-25; Rockland). A subset of cells were coexpressed with constitutively active CaMKII (phosphomimetic mutation of Thr287 to Asp[T287D]), generated as previously described (12). About 300 ng of β_IV_-spectrin plasmid was transfected alone or codelivered with 300 ng CaMKII T287D using 2 μl Lipofectamine 2000 (11668030; Thermo Fisher Scientific), according to the manufacturer's instructions. MS and subsequent analysis was performed by the Proteomics and Metabolomics Laboratory, Lerner Research Institute, The Cleveland Clinic Foundation, Cleveland, OH, USA. The protein samples were fractionated on an SDS-PAGE and submitted for analysis, and a small area around the band was cut from the gel. The gel pieces were washed with water and dehydrated in acetonitrile. The bands were then reduced with DTT and alkylated with iodoacetamide prior to the in-gel digestion. All bands were digested in gel by adding 5 μl of 10 ng/μl trypsin in 50 mM ammonium bicarbonate and incubating overnight at room temperature. The peptides that were formed were extracted from the polyacrylamide in two aliquots of 30 μl 50% acetonitrile with 5% formic acid. These extracts were combined and evaporated to <10 μl in Speedvac and then resuspended in 1% acetic acid to make up a final volume of ∼30 μl for LC–MS analysis. The LC–MS system was a Dionex Ultimate 3000 nano-flow HPLC interfacing with a ThermoScientific Fusion Lumos mass spectrometer system. The HPLC system used an Acclaim PepMap 100 precolum (75 μm × 2 cm, C18, 3 μm, 100 A) followed by an Acclaim PepMap RSLC analytical column (75 μm × 15 cm, C18, 2 μm, 100 A). About 5 μl volumes of the extract were injected, and the peptides eluted from the column by an acetonitrile/0.1% formic acid gradient at a flow rate of 0.3 μl/min were introduced into the source of the mass spectrometer online. The microelectrospray ion source is operated at 2.5 kV. The digest was analyzed using the data-dependent multitask capability of the instrument acquiring full scan mass spectra to determine peptide molecular weights and product ion spectra to determine amino acid sequence in successive instrument scans. The data were analyzed by using all collision-induced dissociation spectra collected in the experiment to search the *Chlorocebus sabaeus* reference sequence protein database and more specifically against the sequence of mouse and human β_IV_-spectrin as expressed in the pcDNA3.1 construct. In order to more accurately measure the relative abundance of the phosphopeptides across these samples, a second LC–MS/MS experiment was performed.

Tandem mass spectra were extracted using the Spectrum Selector node bundled into Proteome Discoverer 2.4 (ThermoFisher). Charge state deconvolution and deisotoping were not performed. The data were analyzed using Proteome Discoverer, version 2.4, with the search engine Sequest. The databases used to search the MS/MS spectra included the Reference Sequence *C. sabaeus* database containing 61,803 entries along with the sequences for mouse Spectrin beta chain (Q62261) and human Spectrin beta chain (Q9H254). An automatically generated decoy database (reversed sequences) was generated for false discovery rate (FDR) calculation. The search was performed looking for fully tryptic peptides with a maximum of three missed cleavages. Oxidation of methionine, acetylation of protein N terminus, and phosphorylation at S, T, and Y were set as dynamic modifications. Carbamidomethylated cysteine was set as a static modification. The precursor mass tolerance for these searches was set to 10 ppm, and the fragment ion mass tolerance was set to 0.6 Da. Peptide and protein identifications were accepted if they could be established at greater than 5.0% probability to achieve an FDR less than 0.1%. Protein identifications were accepted if they could be established at greater than 5.0% probability to achieve an FDR less than 1.0% and contained at least three identified peptides. The positively identified phosphorylated peptides were further subjected to manual inspection with the requirements of a mass measurement less than 5 ppm, and a majority of the ions present were consistent with the identified phosphopeptide. The targeted experiments involve the analysis of specific β_IV_-spectrin peptides, including the phosphorylated and unmodified forms. The chromatograms for these peptides were plotted based on known fragmentation patterns, and the peak areas of these chromatograms were used to determine the extent of phosphorylation.

### Heterologous expression and degradation assays

In order to assess the role of CaMKII activity and β_IV_-spectrin phosphorylation on the stability of β_IV_-spectrin, COS-7 cells (American Type Culture Collection CRL-1651) were transfected with 300 ng of the HA-β_IV_-spectrin pcDNA3.1 construct and 100 ng of constitutively active CaMKII (T287D) or enhanced GFP as previously described (12). Transfections were performed using 2 μl Lipofectamine 2000, according to the manufacturer's protocol. HA-β_IV_-spectrin pcDNA3.1 was also mutated at serine residue 2254 (human βIV-spectrin) as well as additional serine residues 2272, 2305, 2438, and 2560 using the Agilent site-directed mutagenesis kit with primers listed in the following table

**Table:** 

Serine residue	Primer	Sequence
S2254A	Forward	5′-CCTGAGCGGCAAGAGGCAGTCGATCAATCCG-3′
	Reverse	5′-CGGATTGATCGACTGCCTCTTGCCGCTCAGG-3′
S2254E	Forward	5′-TCCTCGGATTGATCGACCTCCTCTTGCCGCTCAGGCC-3′
	Reverse	5′-GGCCTGAGCGGCAAGAGGAGGTCGATCAATCCGAGGA-3′
S2272A	Forward	5′-GAGCGGCAGGAGGCAGCGGAGCACG-3′
	Reverse	5′-CGTGCTCCGCTGCCTCCTGCCGCTC-3′
S2305A	Forward	5′-GAGCGGCAGGAGGCCAGCGAACAGG-3′
	Reverse	5′-CCTGTTCGCTGGCCTCCTGCCGCTC-3′
S2438A	Forward	5′-CTAACCGCAAGTCGGCCAACCGGTCGTGG-3′
	Reverse	5′-CCACGACCGGTTGGCCGACTTGCGGTTAG-3′
S2560A	Forward	5′-AGATCGCAGGGCCGCCGGGCGCAGGAAG-3′
	Reverse	5′-CTTCCTGCGCCCGGCGGCCCTGCGATCT-3′

Underlining indicates mutant bases. Cells were incubated 36 h after transfection, at which time a baseline sample was collected. Remaining wells were then treated with 20 μg/ml CHX in incomplete Dulbecco's modified Eagle's medium (DMEM) to inhibit new protein synthesis. The drug was kept on the cells for 1 and 2 h, at which time cells were collected and lysates prepared for the evaluation of β_IV_-spectrin expression. About 20 μg of protein was loaded into a 10% polyacrylamide gel. An HA-antibody (catalog no. 3724; Cell Signaling) was used to assess levels of β_IV_-spectrin and normalized to the cotransfected CaMKII or GFP to account for variations in transfection efficiency.

### Immunoprecipitation

COS-7 cell pellets were suspended in PhosphoSafe Extraction Buffer (71296; Millipore) supplemented with Protease Inhibitor Cocktail (P8340; Millipore). Whole cell lysates (175 μg) were incubated with HA monoclonal antibody (3724; Cell Signaling) overnight. The antibody-bound proteins were precipitated with TrueBlot Anti-Rabbit Ig IP Agarose Beads (00-8800-25; Rockland), washed with PBS supplemented with protease inhibitor and Halt Phosphatase Inhibitor Cocktail (78420; Thermo Fisher Scientific), and boiled with loading buffer containing 2% β-mercaptoethanol.

### Isolation of primary mouse ventricular CFs

Mouse CFs were isolated from left and right ventricles under sterile conditions, as described (59). Briefly, mouse hearts were minced in 2 mg/ml collagenase II (Worthington) dissolved in 1× Ham's F-10 buffer (Corning). After digestion, the extract was filtered and centrifuged. The supernatant was discarded, and cells were resuspended in DMEM; 1×, supplemented with 10% fetal bovine serum (FBS), 1% l-glutamine, and 1% penicillin/streptomycin. Cells were allowed to adhere to culture plates for approximately 4 to 5 h prior to media removal containing nonadherent cells (*e.g.*, endothelial, myocytes). Fresh feeding media were replenished, and cells were grown for 5 to 7 days to 80% to 100% confluency at 37 °C in 5% CO_2_. All cell experiments were conducted at passage 1.

### Adenoviral generation

Adenoviral β_IV_-spectrin constructs were generated by cloning the HA-tagged β_IV_-spectrin from the pcDNA3.1 construct into the adenovirus construct Ad.CIG (cytomegalovirus promoter combined with internal ribosome entry site expression of enhanced GFP in the second position and β_IV_-spectrin in the first). The resulting adenoviral plasmid was transfected along with the psi5 vector into CRE8 cells for the production and amplification of packaged viral constructs as described here (60).

### CF proliferation assay

CFs were seeded into 12-well culture-treated plates, as described (13). Briefly, cells were adhered for 24 h with serum starvation. At the same time, cells were transduced with adenoviruses (Ad.) Ad.GFP (control), Ad.WT-β_IV_-spectrin, Ad.β_IV_-S2254A, or Ad.β_IV_-S2254E between WT and *qv*^*4J*^ isolated CFs. The next day medium was replaced with 10% FBS/DMEM/penicillin/streptomycin for *qv*^*4J*^ CFs or 2% FBS/DMEM/penicillin/streptomycin for WT cells. For *qv*^*4J*^ CFs, cells were trypsinized at 24, 48, and 72 h postplating. WT CFs were treated 100 nM AngII (1158; R&D Systems) and trypsinized at 24 and 72 h post-AngII stimulation. Cell pellets were resuspended in a fixed volume and manually counted using a hemacytometer to calculate total cell numbers. Accuracy and reproducibility of manual counting was previously confirmed through BrdU proliferation assay (Cell Signaling) (13).

### Collagen gel formation and macroscopic gel contraction measurements

Type I rat-collagen gels (1 mg/ml) were prepared by mixing 10× PBS, sterile water, acidic rat tail collagen, and 1 M NaOH. Cells were added (100,000 cells/ml) and mixed before gelation. Cell-collagen mixtures were cast into 24-well culture plates and incubated at 37 °C in 5% CO_2_ for 45 min. After incubation, 1 ml of culture feeding media was added, and the gels were released from wells. Collagen gels were photographed immediately after release and again the next day (16 h). Photographs were analyzed using NIH ImageJ software (61). Specifically, the diameter of each gel was measured in perpendicular directions and then averaged. As previously described, isotropic compaction was assumed to measure the volume ratio of gels before and after compaction (62).

### Immunoblotting and immunostaining

Primary ventricular CF lysates were prepared by washing cells in PBS and scraping in PhosphoSafe Extraction Buffer (71296; Millipore) supplemented with Protease Inhibitor Cocktail and analyzed using SDS-PAGE and immunoblotting, as described (10, 12, 54). Briefly, equal protein loading was achieved using standard bicinchoninic acid protein assay protocols and verified by Ponceau staining of immunoblots. The following antibodies were used for immunoblotting: HA antibody (for heterologous β_IV_-spectrin) (1:1000 dilution; 3724; Cell Signaling), CaMKII (1:2000 dilution; #A010-56AP; Badrilla), phospho-β_IV_-spectrin (custom antibody generated by GenScript using expression of the peptide sequence RQE{pSRT}VDQPEETARRC for generation in rabbit and affinity purification; 1:1000 dilution), total β_IV_-spectrin (1:1000; MABN1727; Sigma), GFP (1:1000; 2555; Cell Signaling), and GAPDH (1:5000; 10R-G109A; Fitzgerald). Specificity of custom phospho-β_IV_-spectrin antibody was tested using the phosphopeptide used in antibody generation and affinity purification as well as the unmodified antigenic peptide sequence as a peptide blocker. Blocking peptides were used at a concentration of 1.1875 μg/ml by incubating with antibody solution overnight at 4 °C before application on blots. Partial loss of signal with the unmodified antigenic peptide (Fig. S1) and persistence of signal in our S2254A β_IV_-spectrin construct (Fig. 2*A*) suggest some level of reactivity with epitopes adjacent to the phosphorylation site, as expected of polyclonal antibodies. Further evaluation was conducted by pretreatment of the immunoblots with alkaline phosphatase enzyme (Antarctic Phosphatase; NEB #M0289) at 150 units/ml in reaction buffer 10 mM Tris–HCl (pH 8.0 at 37 °C), 5 mM MgCl_2_, 100 mM KCl, 0.02% Triton X-100, and 0.1 mg/ml bovine serum albumin. ImageJ software (61) was used in quantitation of immunoreaction signal.

CFs were seeded for immunostaining in 12-well plates with laminin-coated glass coverslips. Cells were initially seeded in DMEM incomplete media. Experiments using *qv*^*4J*^-derived CFs were treated with adenovirus at the time of plating, and the media changed the next day to 10% FBS/DMEM/penicillin/streptomycin and left another 48 h (72 h culture total). WT-derived CFs were treated with adenovirus at the time of plating and after 24 h were then treated with control or AngII (100 nM) in 2% FBS/DMEM/penicillin/streptomycin for another 24 h. Cells were then fixed in 4% paraformaldehyde and permeabilized with Triton X-100 for 10 min. Nonspecific binding was blocked using blocking buffer comprising 1.5% bovine serum albumin, 3% goat serum in 1× PBS (Invitrogen) overnight at 4 °C. Primary antibodies (β_IV_-spectrin [1:100; Millipore; N393/76], total STAT3 [1:200; Cell Signaling; 4904]) were prepared in blocking buffer and added for overnight incubation at 4 °C. After washing, secondary Alexa Flour 568 antimouse (1:500; Invitrogen; A11004), 488 anti-rabbit (1:500; Invitrogen; A11008), and 633 phalloidin (1:200; Invitrogen; A22284) antibodies were prepared in blocking buffer and incubated for 2 h at room temperature. After washing, 4′,6-diamidino-2-phenylindole (DAPI) (Vecta Laboratories) was applied, and dishes were stored at 4 °C in the dark until they were imaged using confocal microscopy. Images were acquired on a Nikon Ti2 Microscope in a blinded fashion. Multiple random fields were selected from preparation for analysis. STAT3 localization analysis was conducted by identifying nuclear area through DAPI staining and cell body areas through phalloidin staining. Background noise from acquired images was removed using a 3 × 3 median filter in MATLAB (MathWorks). Threshold values were established from these measurements, and binary images were generated. ImageJ software was then used to identify percent signal within DAPI-defined boundaries, relative to total signal within phalloidin-defined boundaries.

### Quantitative real-time PCR

Total RNA from mouse CFs was extracted with TRIzol Reagent following manufacturer's instructions. Multiscribe reverse transcriptase (Invitrogen) was used for the first-strand cDNA synthesis (20 ng/μl) using random primers. Quantitative real-time PCRs (qPCRs) were performed in triplicate on cDNA samples in 96-well optical plates using PowerUp SYBR Green Universal PCR Master Mix (Invitrogen) and a QuantStudio 3 Real-Time PCR System (Applied Biosystems). qPCR data were analyzed using relative standard curve method, and two delta Ct was used to calculate fold changes in relative gene expression. qPCR products were confirmed by melt-curve analysis, amplicon length, and DNA sequencing. Rpl-7 levels were used as a normalization control. Experiments were conducted in technical triplicates. Primers for gene targets are listed herewith.

**Table:** 

Gene name	Primer	Sequence
*Col1a1*	Forward	TCAGCTTTGTGGACCTCCG
	Reverse	GGACCCTTAGGCCATTGTGT
*Fmod*	Forward	AGAAGATCCCTCCTGTCAACAC
	Reverse	GCAGCTTGGAGAAGTTCATGAC
*Serpina3n*	Forward	GGACATTGATGGTGCTGGTGA
	Reverse	CTCTTGCCCGCGTAGAACTC
*Col28a1*	Forward	CCAAAGCCAAACACACTTGC
	Reverse	TCTTCTCACTCAAGCTGTCCAC
*Mmp19*	Forward	TAGATGCTGCCGTTTACTCTCC
	Reverse	AAATCCACATACCGCCACAC
*Serpina3g*	Forward	TGCAATCACAGGAACCAAGG
	Reverse	AACTGCACAACATCCGACAC
*Pdgfra*	Forward	CGCTGACAGTGGCTACATCA
	Reverse	CCTGTCTCGATGGCACTCTC
*Postn*	Forward	AGTAACGAGGCTTGGGAGAAC
	Reverse	CCGTGTTTCAGGTCCTTGGT
*Vim*	Forward	CCAGAGAGAGGAAGCCGAAAG
	Reverse	ACGTGCCAGAGAAGCATTGT
*Rpl7*	Forward	TGGAACCATGGAGGCTGT
	Reverse	CACAGCGGGAACCTTTTTC

### Human tissue

Left ventricular failing heart tissue was obtained from explanted hearts from patients undergoing heart transplantation at The Ohio State University by the Cardiac Transplant Team. Left ventricular tissue from nonfailing donor hearts (without a history of cardiac dysfunction) not suitable for transplantation was obtained through the Lifeline of Ohio Organ Procurement Organization. All samples were flushed with ice-cold cardioplegic solution and flash frozen within 45 min of removal and kept frozen at −80 °C. Frozen samples were ground before treatment with PhosphoSafe Extraction Reagent (71296; Millipore) supplemented with protease inhibitor (P8340; Sigma) and followed up by sonication to facilitate homogenization. The local institutional review board approved the use of human subject tissue. This investigation conforms to the principles outlined in the Declaration of Helsinki. Human hearts used in this study were deidentified and labeled with six digit random codes for reference to clinical descriptions.

### Statistics

SigmaPlot 14.0 (SYSTAT) was used for statistical analysis. A 2-tailed *t* test was used to determine *p* values for single comparisons. For multiple comparisons, a one-way ANOVA with Tukey honest significant difference post hoc test was used (data presented as mean ± SEM). The null hypothesis was rejected for *p* value <0.05.

## Data availability

The MS proteomics data have been deposited to the ProteomeXchange Consortium *via* the PRIDE (63) partner repository with the dataset identifier PXD025776 and 10.6019/PXD025776. All remaining data are contained within the article.

## Supporting information

This article contains supporting information.

## Conflict of interest

The authors declare that they have no conflicts of interest with the contents of this article.
