# Supplementary material for: Ca2+/calmodulin kinase II–dependent regulation of βIV-spectrin modulates cardiac fibroblast gene expression, proliferation, and contractility
Source: J Biol Chem. 2021 Jun 18;297(1):100893. doi: 10.1016/j.jbc.2021.100893 (PMC8294584; doi:10.1016/j.jbc.2021.100893)
Supplement: Supplemental Figures S1–S4 and Table S1 [file mmc1.pdf]

Ca^{2+}/calmodulin kinase II–dependent regulation of β_{IV}-spectrin modulates cardiac fibroblast gene expression, proliferation, and contractility

Drew M. Nassal[1,2], Nehal J. Patel[1,2], Sathya D. Unudurthi[1], Rebecca Shaheen[1,2], Jane Yu[1,2], Peter J. Mohler[1,3,4], Thomas J. Hund[1,2,4*]

Supporting information–Supplemental Figures 1-4 and Table 1 with legends

Fig S1

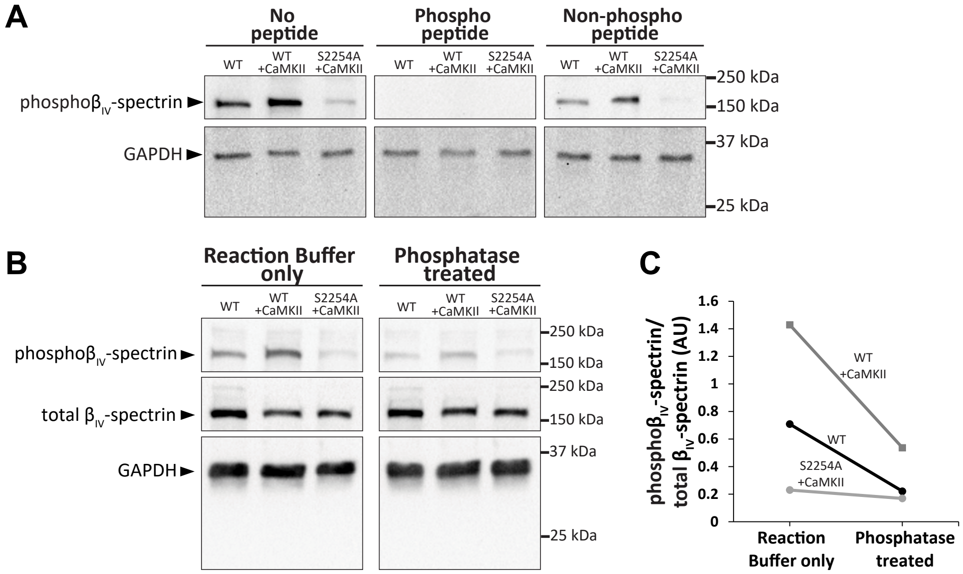

Supplemental Figure 1. Evaluation of phosphoβ_{IV}-spectrin antibody. *A*, Immunoblots for phosphorylated β_{IV}-spectrin using a novel polyclonal antibody recognizing phospho-β_{IV}-spectrin(S2254). Lysate were prepared from COS7 cells transfected for 48 hrs with WT β_{IV}-spectrin ± CaMKII T287D, or phospho-ablated β_{IV}-spectrin (S2254A) with CaMKII T287D. Antibody solution was pre-incubated without immunogenic peptide, with the phospho-specific immunogenic peptide, or the unmodified peptide before exposure to blots. *B*, Same conditions as evaluated in *A*, except prior to membrane blocking and antibody exposure, the membrane was treated with buffer alone or buffer with Antarctic Phosphatase for β_{IV}-spectrin dephosphorylation for 2 hrs at 37°C with gentle rocking every 15 minutes. *C*, Quantification of immunoblot featured in panel B.

Fig S2

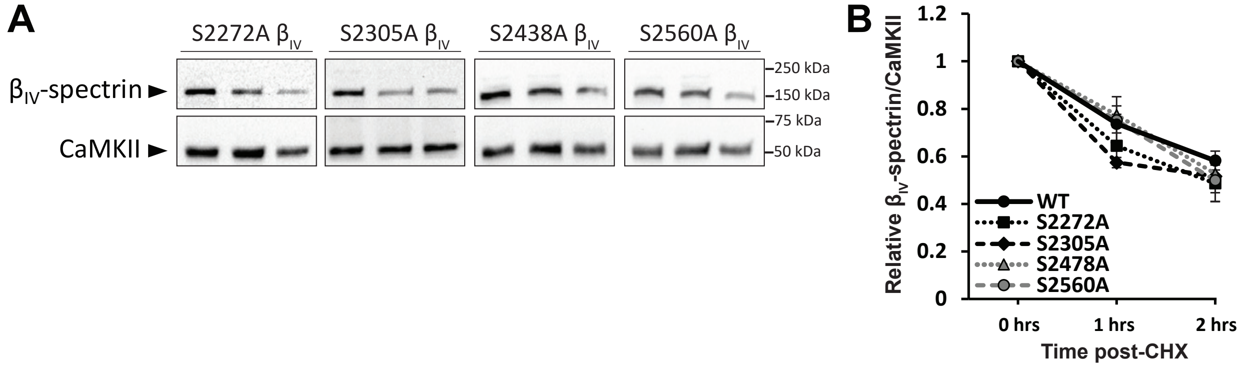

Supplemental Figure 2. Evaluation of alternative predicted CaMKII phosphorylation residues in the C-terminus of β_{IV}-spectrin and their impact on degradation. *A*, Representative immunoblots and *B,* densitometric measurements from COS7 cells transfected with WT or alternative phospho-ablated β_{IV}-spectrin constructs S2272A, S2305A, 2438A, or 2560A co-expressed with constitutively active CaMKII, T287D. Cells were cultured for 48 hrs after which degradation assays were performed by treating COS7 cells with CHX (20 µM) to stop new protein synthesis for 1 and 2 hrs to measure the rate of β_{IV}-spectrin loss. β_{IV}-spectrin expression was normalized against co-transfected CaMKII to also account for transfection control.

Fig S3

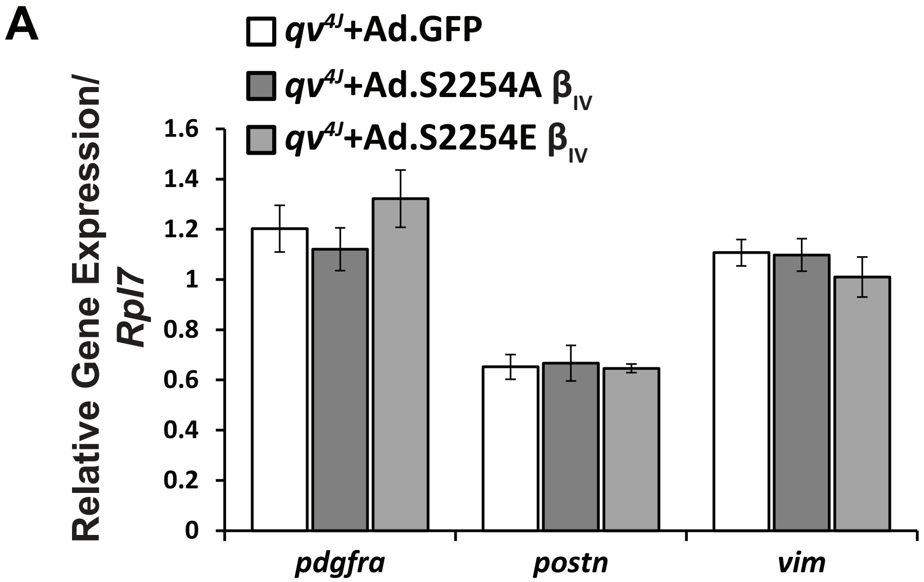

Supplemental Figure 3. Evaluation of negative control CF gene targets not regulated by β_{IV}-spectrin phosphorylation status. *A*, Summary data for expression of fibrotic associated genes (relative to *Rpl7*) evaluated by quantitative PCR from *qv*^{4J} CFs 72 hrs after transduction with Ad.GFP, Ad.β_{IV}-S2254A, or Ad.β_{IV}-S2254E.

Fig S4

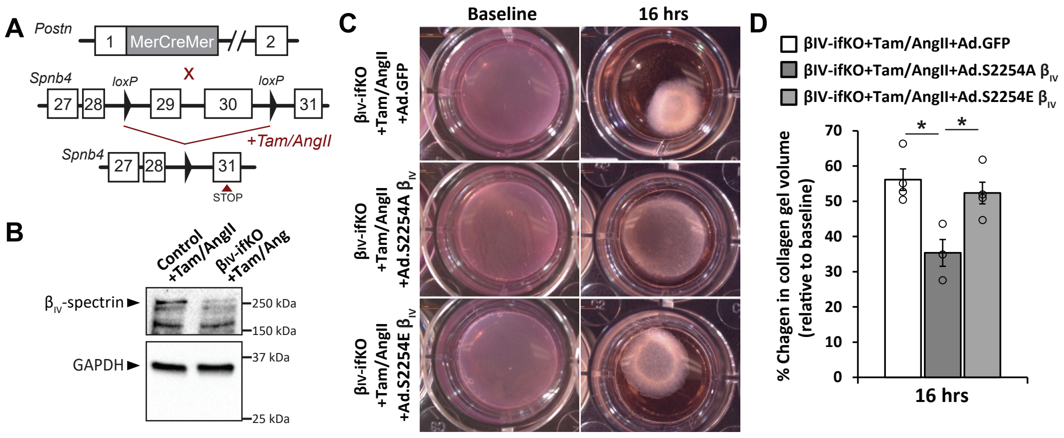

Supplemental Figure 4. Fibroblast-specific deletion of β_{IV}-spectrin and restoration with phospho-ablated/mimetic β_{IV}-spectrin differentially regulates CF collagen gel compaction. *A*, Schemtic describing β_{IV}-spectrin floxed mice cross-bred with periostinMerCreMer mice to generate an inducible fibroblast-specific β_{IV}-spectrin KO mouse (β_{IV}-ifKO). 7-day treatment of animals with angiotensin II (2.16 mg/kg i.p. injection daily) together with tamoxifen (75 mg/kg i.p. injection daily) (+Tam/Ang II) was used to activate periostinMerCreMer (periostin highly expressed in activated fibroblasts) and effective β_{IV}-spectrin KO in CFs. *B*, Representative immunoblots from cultured CF lysates prepared from control (β_{IV}-floxed, Cre-) and β_{IV}-ifKO mice. *C*, Representative images of collagen gels seeded with β_{IV}-ifKO CFs expressing Ad.GFP, Ad.β_{IV}-S2254A, or Ad.β_{IV}-S2254E just after collagen gel formation and release (Baseline) and 16 hrs after incubation. *E,* Summary data of the change in collagen gel volume over the course of 16 hrs in Ad.GFP, Ad.β_{IV}-S2254A, or Ad.β_{IV}-S2254E conditions. *$P < 0.05$ by one-way ANOVA followed by Tukey HSD test ($F = 7.56$, $p = 0.0143$; $n \geq 3$.

Table S1

Spectrin peptides identified (34 peptides, 31% of the protein sequence)										
Protein Accession	[M+H]	m/z	z	Δm ppm	Missed Cleavages	Ret Time min	Sequence	Modifications	Xcorr	ΔCn
Q62261	1403.674	468.5636	3	1.81	0	36.172	HQAFMAELAQNK	1xOxidation [M5]	1.61	0
Q62261	2817.443	705.119	4	3.87	0	51.2533	SAPAQGGSAPAPPPPPTHTVQHEGFLLR		2.35	0
Q62261	1113.601	557.3062	2	3.53	0	34.0504	QLQEGAAQLR		2.54	0
Q62261	1388.629	694.8189	2	1.15	0	27.8549	QESVDQPEETAR		2.29	0
Q62261	1551.711	776.3626	2	4.61	0	50.0671	QCWAELESTTQAK	1xCarbamidomethyl [C2]	2.74	0
Q62261	2724.351	908.7922	3	4.07	0	62.0203	LLHMESQLQDVDPGGDLATVNSQLK	1xOxidation [M4]	2.11	0
Q62261	997.5677	499.2888	2	2.7	0	46.535	LLEPLQER		2.06	0
Q62261	1169.627	585.3196	2	3.91	0	38.38	LHVSSTADALR		3.03	0
Q62261	2089.017	697.0131	3	3.68	1	53.6709	HQAFMAELAQNKEWLEK	1xOxidation [M5]	2.83	0
Q62261	1372.689	686.8482	2	0.15	0	48.8034	VPELATCQELGR	1xCarbamidomethyl [C7]	3.03	0
Q62261	1279.712	427.2434	3	2.95	0	38.6482	GTGLQAVQQHIK		0.95	0
Q62261	1359.618	680.3149	2	3.73	0	42.7146	FSEFASETGTAGR		3.93	0
Q62261	1812.928	906.9677	2	0.13	0	77.7371	VSLEQQYWLYQLSR		3.97	0
Q62261	1457.785	729.3991	2	4.27	0	80.4356	ATLADIVEQLQEK		3.8	0
Q62261	1147.541	574.2755	2	2.12	0	39.6882	ELLAACEDAR	1xCarbamidomethyl [C6]	2.24	0
Q62261	1573.807	787.4104	2	4.28	0	73.7654	ELGSSVDEVEQLIR		3.6	0
Q62261	1929.97	643.9962	3	2.1	0	41.068	EGQQLMQEKPELAASVR	1xOxidation [M6]	3.46	0
Q62261	1642.891	821.9518	2	3.14	0	62.0062	EAGPGIPAGVPSLPQPR		2.37	0
Q62261	1933.932	645.3174	3	2.68	0	97.6473	DGLNEAWAELLELMGTR	1xOxidation [M14]	2.9	0
Q62261	1654.891	552.3042	3	4.18	0	73.1332	AFEHDLQLLVSQVR		2.77	0
Q62261	786.4832	393.7467	2	3.71	0	45.6815	AGVLASLR		2.7	0

Q62261	1468.595	734.8032	2	2.65	0	32.7226	QESVDQPEETAR	1xPhospho [S3]	2.74	0
Q62261	858.4316	429.7208	2	3.19	0	21.5256	SPEAEAVR		1.99	0
Q62261	903.4319	452.221	2	3.2	0	31.9466	AAAAWEER		2.34	0
Q62261	1073.547	537.2788	2	2.69	0	29.877	ELQGQIEEK		1.85	0
Q62261	829.489	415.2495	2	3.21	0	32.2407	AQLLAASR		1.9	0
Q62261	2852.446	713.8696	4	3.8	1	57.6088	LLHMESQLQDVDPGGDLATVNSQLKK	1xOxidation [M4]	1.99	0
Q62261	1070.5	535.7549	2	2.4	0	25.9043	ATSEVASDYK		2.41	0
Q62261	822.4356	411.7229	2	3.64	0	33.8997	QLFEASK		1.94	0
Q62261	1074.561	537.7867	2	4.54	0	34.8909	LPLAMQTER	1xOxidation [M5]	1.49	0
Q62261	1198.595	599.8032	2	3.37	1	14.832	ATSEVASDYKK		1.53	0
Q62261	1204.628	602.82	2	3.9	1	31.6968	RASDTLSAEVR		2.23	0
Q62261	1195.599	598.3055	2	3.61	0	59.4945	FFGDPTELAAK		1.89	0
Q62261	1969.029	657.0179	3	5.21	1	69.7918	RVSLEQQYWLYQLSR		1.99	0

Supplemental Table 1. List of identified β_{IV}-spectrin peptides and associated characteristics from mass spectrometry dataset.
